# *Arctostaphylos uva-ursi* L. leaves extract and its modified cysteine preparation for the management of insulin resistance: chemical analysis and bioactivity

**DOI:** 10.1007/s13659-022-00352-1

**Published:** 2022-08-12

**Authors:** Ganna Kravchenko, Oksana Krasilnikova, Ain Raal, Matar Mazen, Natalia Chaika, Igor Kireyev, Andriy Grytsyk, Oleh Koshovyi

**Affiliations:** 1 National University of Pharmacy, 53 Pushkinska Str., Kharkiv, 61002 Ukraine; 2 Institute of Pharmacy, Faculty of Medicine, University of Tartu, Nooruse 1, 50411 Tartu, Estonia; 3 Ivano-Frankivsk National Medical University, 2 Halytska Str., Ivano-Frankivsk, 76018 Ukraine

**Keywords:** *Arctostaphylos uva-ursi* L., Extract, Hypoglycaemic activity, Leaves, Phenolic substances

## Abstract

Bearberry (*Arctostaphylos uva-ursi* L.) is a perennial plant of the heather family (*Ericaceae*). The leaves are dominated by arbutin, phenol carbonic acids flavonoids, saponins, etc. It was previously shown that the bearberry leaves extract reduced blood glucose level in healthy animals under glucose overload, so it need to be studied more detail. The aim of the study was to investigate the chemical composition and the effect of dry alcohol extract from bearberry leaves, which enriched with cysteine, on the rats pancreas under experimental dexamethasone-induced insulin resistance (IR). *Arctostaphylos uva-ursi* L. leaves extract and its modified cysteine preparation were obtained according to the developed method with 50% ethanol solution. Their phytochemical profile, hypoglycaemic and pancreatic protective effect were investigated. Phenologlycoside (arbutin), phenolic carboxylic acid (gallic acid), 5 flavonoids and 4 hydroxycinnamic acids were identified and quantified in the extracts by HPLC. Present data revealed that bearberry leaves alcoholic dry extract enriched with cysteine has a hypoglycaemic and pancreatic protective effect in treated animals under dexamethasone-induced IR model. Treatment improved hyperglycaemia, insulin resistance and beta cell reduction induced by dexamethasone injections.

**Graphical Abstract:**

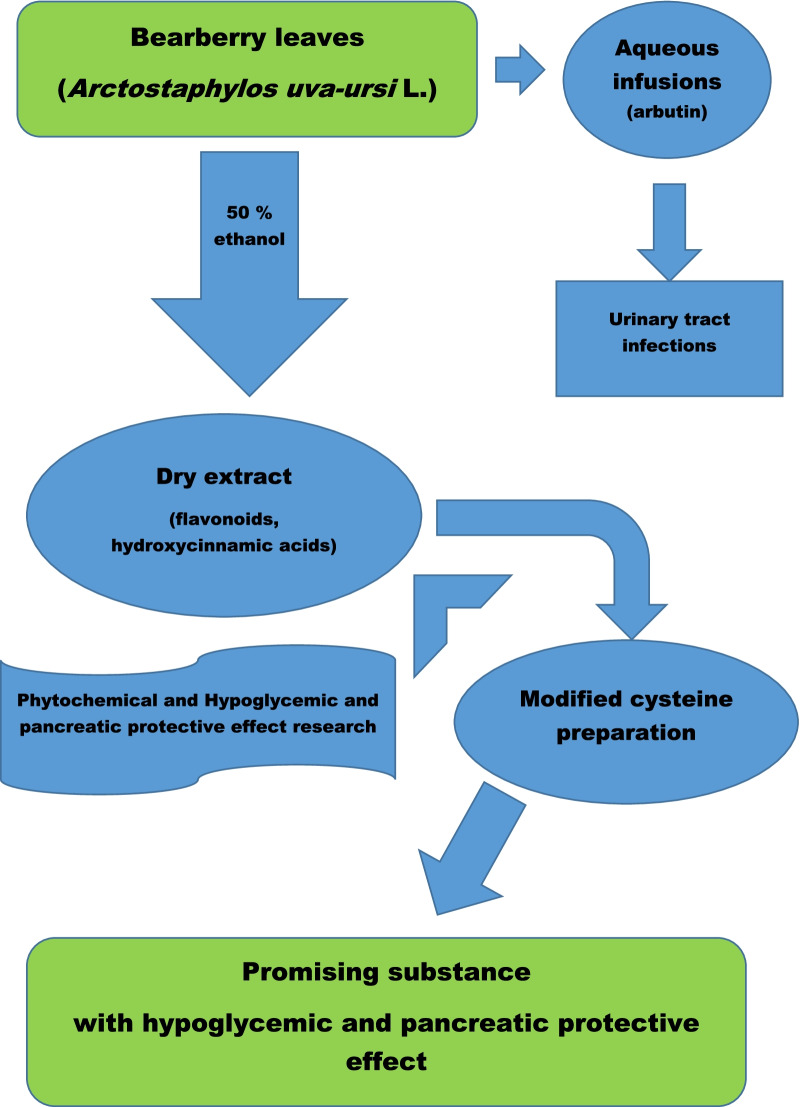

## Introduction

The problem of diabetes mellitus type 2 (T2DM) due to its prevalence, severity and complications is one of the most pressing in the world [1]. T2DM is a metabolic disease characterized by chronic hyperglycaemia, which caused by deterioration of insulin interaction with tissue cells [2]. Assumed mechanism for the T2DM development is insulin resistance (IR), which is precisely a decrease in the biological response of cells to one or more effects of insulin under its normal concentration in the blood [3]. Healthy pancreatic beta cells can adapt to decreased tissue insulin sensitivity and prevent the fasting hyperglycaemia by increasing insulin secretion, but under the pre-diabetic state and T2DM, the rate at which glucose enters the bloodstream exceeds the rate of glucose uptake by tissues. However, we can find different points of view regarding the role of pancreatic beta cells function, as well as insulin resistance (IR) development in the T2DM pathogenesis [4]. Anyway, according to modern ideas, T2DM development is the result of progressive impairment of the pancreatic beta cells functional activity under long-term IR.

The central role of beta cells dysfunction in the T2DM pathogenesis regardless of antidiabetic therapy has been confirmed by some clinical studies [5]. And, no doubt, scientists with practicians tried to “combine” the theory with searching for the possible ways of treatment. Surely, a number of synthetic medications, which are used to treat patients with T2DM, have been developed and are produced all over the world. Modern therapy that aims to monitor glucose level includes the following mechanisms: reduction of insulin secretion from pancreatic beta cells, stimulation of glucagon secretion from pancreatic alpha cells, elevation of liver glucose production, lipolysis enhancement, attenuation or diminution of glucose uptake in peripheral tissues such as skeletal muscle, liver, and adipose tissue, etc. [6]. However, synthetic medications have been shown to have a number of side effects, including the activation of free radical oxidation processes [7]. Today, herbal medicine is becoming an important part of the treatment and prevention of metabolic disorders. It can be used in certain stages of T2DM as monotherapy in combination with diet therapy, as well as in combination with antihyperglycemic agents. It has been studied that plant glycosides and flavonoids can have hypoglycaemic and antioxidant effects [8].

It has previously been shown that blueberry (*Vaccinium myrtillus* L.) [9, 10] and highbush blueberry (*Vaccinium corymbosum* L.) [11] extracts have the ability to lower glucose levels and adjust lipid levels in a model of induced IR in rats. These extracts have been shown to be promising agents for the metabolic syndrome correction.

Bearberry (*Arctostaphylos uva-ursi* L.) is a perennial plant of the heather family (Ericaceae). The leaves are dominated by arbutin, phenol carbonic acids flavonoids, saponins, etc., and which is used in aqueous infusions for the treatment of different diseases [12]. It was proved in our experiments [13, 14] that extracts obtained from bearberry leaves reduce blood glucose level in healthy animals under glucose overload and in animals with modelled pathology [15]. The dry extract of *Arctostaphylos uva-ursi*, modified with phenylalanine, also showed pronounced diuretic, anti-inflammatory and antimicrobial (in relation to *St. aureus, E. coli, P. vulgaris, P. aeruginosa, B. subtilis and C. albicans*) activities [16].

Because existing T2DM pharmacotherapy not always prevent beta cell function disorders, which inevitably leads to impaired glycaemic control and progression of diabetic micro- and macrovascular complications, the search for new pharmacological agents that can preserve endocrine pancreatic function is especially relevant.

Modification of biologically active molecules by their conjugation with amino acids is a known strategy for modification of their biological properties. For example, the synthetic medicine Valtrex was created by combining acyclovir with the amino acid valine [17], the medicine l-lysine escinate was created using the modification of the complex of chestnut triterpene saponins (β-escin) with l-lysine [17, 18]. Therefore, it is interesting to use such modification the total plant extract for improvement of its biological properties. It was previously shown that the modification of *Leonurus cardiaca* L. tincture with amino acids has led to the creation of new more active substances with anxiolytic activity [19]. The modification of the blueberry and highbush blueberry leaves extracts with arginine allowed to create a substance with pronounced hypoglycemic and hypolipidemic activities [9, 11]. The modification of the extract of bearberry leaves with phenylalanine allowed to create a substance with a pronounced diuretic and anti-inflammatory effect [16]. All of this indicates the viability of the chosen direction.

Several previous studies showed that amino acids present in proteins can interact with polyphenols including chlorogenic acid and flavonoid derivatives. The interaction often results in non-covalent binding of NH_2_ groups of amino acids and OH groups of polyphenols. It was also reported that in some cases covalent adducts including Schiff bases can be formed [20, 21].

It was previously shown that the bearberry leaves extract modified with cysteine mostly reduced blood glucose level in healthy animals under glucose overload (OGTT) [13, 14], so it needs to be studied more detail.

The aim of the current study was to investigate the chemical composition and the effect of dry alcohol extract from bearberry leaves, which enriched with cysteine, on the rats pancreas under experimental dexamethasone-induced IR.

## Results and discussion

### Chemical analysis

The bearberry leaves extracts are light brown powders with a specific odour.

TLC analysis of simple phenols on the chromatogram in daylight revealed brown areas at the level of gallic acid zones and red spots at the level of the arbutin zone. Thus, gallic acid and arbutin were found in the bearberry extracts. TLC analysis of hydroxycinnamic acids and flavonoids in the extracts revealed at least 3 compounds, of which compared to standard samples were identified caffeic and chlorogenic acids, and at least 5 substances of flavonoids, from which identified rutin and hyperoside. On TLC chromatograms of the extracts in daylight, identified red areas at the level of the catechin zone, at the level of the epigallocatechin zone—no zones were detected. Thus, catechin was detected in the bearberry extracts. Alkaloids weren’t identified in the extracts.

**Fig. 1:**
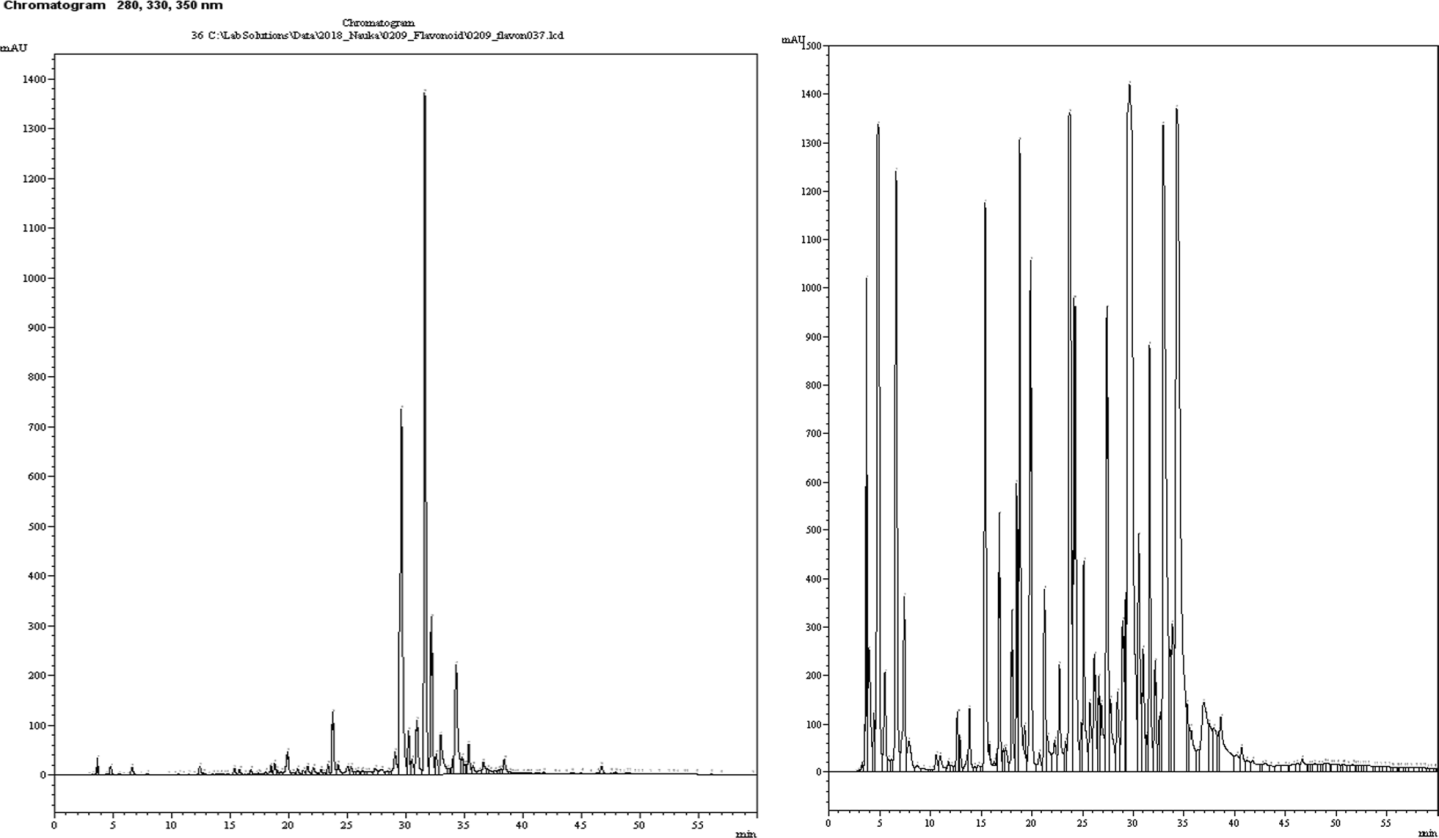
Typical chromatograms of the bearberry extract (PE50)

The content of the main BAS of phenolic nature was determined by HPLC and spectrophotometry in the obtained bearberry leaves extracts (Table 1; Fig. 1).

**Table 1:** The results of HPLC and spectrophotometry analysis of the dry bearberry leaves extracts

Substance	Content of the substance		
	PE	PEC	Decrease, %
Arbutin, mg/100 g	2943.67 ± 52.82	2672.89 ± 62.11	9.20
Gallic acid, mg/100 g	149.12 ± 4.64	135.87 ± 3.32	8.89
Caffeic acid, mg/100 g	87.09 ± 3.51	78.62 ± 2.71	9.73
p-Coumaric acid, mg/100 g	42.14 ± 2.83	37.98 ± 2.24	9.87
Chlorogenic acid, mg/100 g	214.38 ± 2.92	195.11 ± 3.57	8.99
Protocatechuic acid, mg/100 g	14.13 ± 1.10	12.77 ± 1.62	9.62
Rutin, mg/100 g	11.68 ± 0.05	10.57 ± 0.04	9.50
Hyperoside, mg/100 g	438.12 ± 11.38	409.51 ± 10.54	6.5
Quercitrin, mg/100 g	13.79 ± 0.47	13.08 ± 0,42	5.15
Quercetin, mg/100 g	3.49 ± 0.01	3.13 ± 0.04	10.32
Catechin, mg/100 g	268.91 ± 5.23	249.53 ± 6.13	7.21
The sum of hydroquinonederived compounds (in terms of arbutin), %	6.57 ± 0.08	6.35 ± 0.06	3.35
Hydroxycinnamic acids (in terms of chlorogenic acid), %	2.71 ± 0.02	2.47 ± 0.04	8.86
Flavonoids (in terms of rutin), %	4.34 ± 0.06	3.97 ± 0.047	8.53
The sum of phenolic compounds (in terms of gallic acid), %	17.12 ± 0.07	15.61 ± 0.11	8.82

Phenologlycoside (arbutin), phenolic carboxylic acid (gallic acid), 5 flavonoids and 4 hydroxycinnamic acids were identified and quantified in the bearberry extracts by HPLC. Hyperoside and catechin were dominant among flavonoids, and caffeic and chlorogenic acids were dominant among hydroxycinnamic acids. The chromatograms of the modified extract PE50_cys in this system did not change significantly, but the area of the detected substances peaks decreased. The content of all identified phenolic compounds in the modified extracts was lower from 3.35 to 10.32% compared to the native extract (PE50) obtained with a 50% ethanol solution, because 3.63 g cysteine had been added. As for the identified phenolic compounds, they have previously been found in the extract from this raw material, but their presence and quantity will be crucial in the future in the development of quality control methods.

When dissolving the dry modified bearberry extract PE50_cys in water, a clear dark brown solution is formed, in contrast to the extract PE50 obtained with 50% ethanol, in which there is both opalescence and a small amount of sediment. This indicates that the solubility of phenolic compounds increases with the addition of cysteine due to the formation of more hydrophilic conjugates and complexes. In addition, from the general UV spectra of the extracts it can be seen that within the spectrum of aromatic groups there are hypo- and hyperchromic shifts, which also indicates the formation of conjugates and complexes. But these complexes are not stable, because by HPLC chromatographed in an acidic environment, they are not detected.

The content of hydroquinone-derived compounds (in terms of arbutin), hydroxycinnamic acids (in terms of chlorogenic acid), flavonoids (in terms of rutin) and the number of phenolic compounds (in terms of gallic acid) was determined in the obtained extracts by spectrophotometry. As can be seen from the obtained results, in the modified extract the content of all these groups of BAS is lower than the extract from the broth, while their hypoglycaemic effect exceeds the extract obtained with 50% ethanol solution. This suggests that the addition of cysteine potentiates the activity of phenolic compounds of bearberry leaves.

### Pharmacological activity

Low-dose dexamethasone injections have been used in different modifications to induce IR and T2DM during last decades [22]. To assess whether long-term dexamethasone injections in current modification induce IR, we determined FBG, IRI and recalculated HOMA indices (Table 2).

**Table 2:** Effect of dry bearberry leaves extracts on HOMA-IR in a model of insulin-resistance induced by dexamethasone injections in rats

Indices	Time period									
	Initial	5 weeks		7 weeks/experimental groups						
		IC	Dex	IC	Dex	Dex_PE50	Dex_PE50_cys	Dex_cys	Dex_met	Dex_arph
Glucose, mmol/L	4.15 ± 0.28	4.03 ± 0.41	5.93 ± 0.63*	3.95 ± 0.27	7.58 ± 0.91*	5.81 ± 0.54*/#	5.37 ± 0.49#	6.01 ± 0.47*	5.33 ± 0.31*/#	6.11 ± 0.47*
IRI, pmol/L	73 ± 7	72 ± 8	105 ± 8*	74 ± 9	121 ± 15*	91 ± 9*	82 ± 7*/#	90 ± 9*	71 ± 6*/#	95 ± 10
HOMA	1.9 ± 0.6	1.8 ± 0.6	4.0 ± 0.8*/#	1.7 ± 0.6	5.9 ± 0.9	3.4 ± 0.6*/#	2.8 ± 0.3#	3.5 ± 0.2	2.4 ± 0.2	3.7 ± 0.4

Each value represents the mean ± standard error (n = 6)

^*^Indicates significant difference relative to IC group (p ≤ 0.05)

^#^Indicates significant difference relative to Dex group (p ≤ 0.05)

#### Relative pancreas weight

**Fig. 2:**
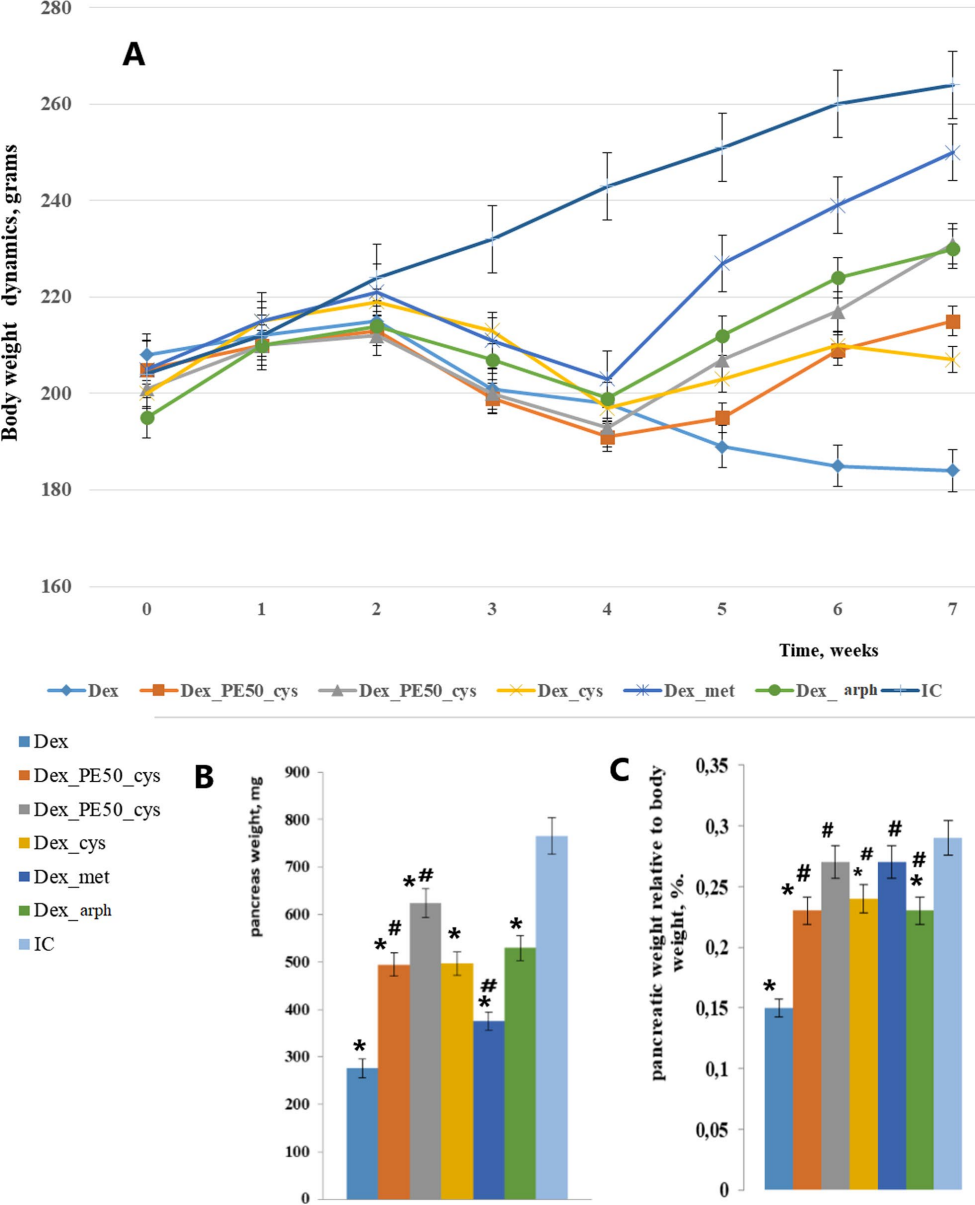
**a** Body weight dynamics, grams (ordinate axis) per weeks (abscissa axis). **b** Pancreas weight, grams. **c** Pancreatic weight relative to body weight, %. Each value represents the mean ± standard error of (n = 6). *indicates significant difference relative to IC group (p ≤ 0.05). #indicates significant difference relative to Dex group (p ≤ 0.05)

The mean body weight of IC rats by the end of 7th week was substantially higher than that of Dex rats; as, however, during the whole experiment. Thus, significant differences in body weight were recorded as early as the 3rd week (Fig. 2a). Administration of studied compounds gives the opportunity to animals to gain body weight according to their age. The weight of rat pancreas was the object of our interest. As it turned out, the absolute weight of pancreas in different experimental groups varied significantly (Fig. 2b). But what we are really interesting is the ratio between the pancreas weight and body weight. Thus, the relative organ weight of IC animal pancreas is significantly higher than of Dex ones. After the 2-weeks-administration of novel obtained extracts and reference medications, the relative weight of pancreas increased but didn’t reach this index in healthy animals (Fig. 2c).

#### Antioxidant status and oxidative stress markers

As shown in Table 3, a hyperglycaemic state induced by dexamethasone long-term injections led to an increase in the levels of oxidative markers such as lipid-peroxidation products measured as TBARS; products such as DC and also lower the activity of antioxidant enzymes.

**Table 3:** Effect of dry bearberry leaves extracts on some antioxidant parameters and JNK content in a model of insulin-resistance induced by dexamethasone injections in rats

Experimental groups	Indices							
	TBARS, nmol/mg protein	DC, nmol/mg protein	GSH, nmol/mg tissue	CAT, µmol/mg protein	SOD, U/mg protein	GPx, U/mg protein	Total JNK, ng/mg protein	p-JNK, ng/mg protein
IC	99 ± 11	47 ± 7	82 ± 7	0.54 ± 0.03	28.3 ± 1.7	0.68 ± 0.06	274 ± 19	73 ± 8
Dex	148 ± 21*	93 ± 8*	36 ± 6*	0.33 ± 0.04*	16.1 ± 0.9*	0.44 ± 0.05*	385 ± 21*	124 ± 12*
Dex_PE50	112 ± 14	64 ± 4*/#	60 ± 7#	0.41 ± 0.05*/#	21.3 ± 1.5	0.51 ± 0.07	330 ± 28	112 ± 9*
Dex_PE50 _cys	103 ± 15#	59 ± 6#	73 ± 8#	0.51 ± 0.05#	23.7 ± 1.7#	0.63 ± 0.03#	301 ± 15#	84 ± 6#
Dex_cys	129 ± 16*	71 ± 6*/#/&	45 ± 9*/&	0.40 ± 0.02*/#	19.9 ± 2.3*	0.56 ± 0.08	311 ± 12*/#	107 ± 11*/&
Dex_met	114 ± 12#	53 ± 8#	70 ± 6#	0.48 ± 0.06#	24.6 ± 1.4	0.61 ± 0.07#	299 ± 14#	91 ± 8
Dex_arph	126 ± 23	69 ± 7*	56 ± 7*/#	0.37 ± 0.03*	22.3 ± 1.7*	0.59 ± 0.04	324 ± 31	114 ± 10*

Each value represents the mean ± standard error (n = 6)

^*^Indicates significant difference relative to IC group (p ≤ 0,05)

^#^Indicates significant difference relative to Dex group (p ≤ 0.05)

^&^Indicates significant difference relative to Dex_PE_cys group (p ≤ 0.05)

The experimental pathology development was accompanied by activation of lipid peroxidation and antioxidant defence system depletion in the pancreatic cells. Thus, it was found that in the pancreatic tissue homogenates from Dex group animals, the content of both primarily and end products of lipid peroxidation—CD and TBARS were increased by 1.97 and 1.49 times, respectively. The GSH level decreased by 2.27 times, which is apparently due to its use in the process of ROS inactivation, as well as by GSH-Rx activity decrease (Table 3).

Numerous experimental evidences proved oxidative stress development as one of the consequences of chronic hyperglycaemia [23]. Particularly, experiments both in vivo and in vitro using pancreatic beta cells culture, also provided evidence for an increase in ROS production in pancreas [24]. In this regard, it is important to evaluate indices that indicate the oxidative stress development and cell antioxidant defence system answer. Our findings of the current experiment that revealed the accumulation of TBARS and DC in pancreas homogenate at the end of 7th week of low dexamethasone administration proved the intensification of ROS formation.

It is known that JNK is primarily activated due to the ROS formation. We found that in the pancreatic tissue of rats from the Dex group, both the total JNK content and its phosphorylated form—p-JNK were increased by 1.4 and 1.69 times, respectively. The activity of the first line enzymes of the antioxidant (AO) defence—CAT and SOD—decreased by 1.5 and 1.75 times, respectively. PE50_cis administration to animals with control pathology inhibited lipid peroxidation processes and improved the AO protection of pancreatic cells. Thus, the DC and TBARS contents were decreased by 1.57 and 1.44 times. The GSH level was restored almost to the initial values, which, apparently, was caused not only by the oxidative stress suppression, but also by the GSH-Rx activation. The CAT and SOD activities increased by 1.5 and 1.43 times. The total JNK level decreased by 1.28 times, but did not reach the initial values, while the content of p-JNK was practically normalized (Table 3).

The total JNK level increase as a result of long-term dexamethasone administration might be due to glucocorticoid effects on the MAP kinases expression [25]. In our experiment, the IR development was proved in Dex group animals with the help of generally accepted methods—hyperglycaemia and hyperinsulinemia (Table 2) and is accompanied by oxidative stress development in rat pancreas, JNK activation and an increase in the p-JNK content (Table 3).

There is evidence concerning the probable harmful effects of JNK activation in beta cells, which can even cause the deep cell damage [26]. JNK activation in excretory cells is a key link for the diabetes of the exocrine pancreas, which leads to cell death, inflammatory processes activation, pancreatitis, etc. [27]. Thereby, in our study it was revealed in a decrease in relative to body weight pancreatic weight (Fig. 2). With a high degree of probability, JNK activation in pancreas tissue led to morphological changes find in histological samples. JNK phosphorylation leads to its translocation into mitochondria, interaction with Sab protein, inhibition of tissue respiration and release of reactive oxygen species, that is the formation of a JNK-Sab-ROS activation loop [28].

PE50_cis administration to animals significantly reduces the level of pJNK, inhibits lipid peroxidation, and improves the activity of antioxidant defence enzymes (Table 3). The observed effect is mediated by the combined action of several components. Thus, lipid peroxidation suppression is a result of polyphenolic components antioxidant activity, among which arbutin has a pronounced activity, moreover, gallic acid inhibits JNK signal transduction. The increased GSH level is maintained by presence of the amino acid cysteine, which also contributes to an increase in the activity of enzymes containing SH-groups in the active site [29].

Thus, we supposed that the recent combination of plant polyphenols of the novel extract and the amino acid cysteine prevents lipid peroxidation, restores the antioxidant defence system, and destroys the JNK-SAB-ROS loop, which, in turn, restores the structure and improved altered functional beta cells activity under dexamethasone induced IR.

Moreover, current studies show that administration of PE50_cys compounds antioxidant properties led to evaluation of oxidative stress lowering of TBARS and increase of SOD and CAT activities.

Pancreatic cells are highly sensitive to oxidative stress, ROS accumulation can lead to decrease in beta-cells mass. It is important to mention that JNK pathway plays the key role in beta-cells dysfunction and insulin resistance, since oxidative stress triggers stress-activated protein kinases (SAPKs), JNK and p38 group of mitogen-activated protein kinases (p38 MAPK) phosphorylation. Thus, JNK could be a potential therapeutic target for treating so cold “glucose toxicity” found in diabetes.

#### Study of histopathological changes in the pancreas

As shown by optical examination in intact rats, the gland exocrine part is presented by lobes, between which are seen very narrow connective tissue membranes. As for pancreatic islets, or islets of Langerhans (Li), they are clearly separated from the surrounding exocrine parenchyma; consist of a set of light polygonal cells strands. Common sinusoidal capillaries are sometimes visible between the strands of cells. The islet cells bulk (central part) consists from beta cells. Alpha cells are located as a chain on the periphery of the island (Fig. 2). It should be noted that this zonal distribution of alpha and beta cells is typical for this species and belongs to the “cloak” type of islands.

According to the morphometry results, the Li optical density in rats of the intact control group was equal to 19.83, however, dexamethasone injections significantly decreased this index. Their size ranging from large to small ones were distributed as percentage, and in untreated IR rats group this ratio changed compared with IC animals leading to an increase in the proportion of small ones. (Table 4). Functional index also shifts downward significantly. Administration of PE_cys and reference medications improve the situation, though, the most pronounced effect was shown by metformin.

**Table 4:** Morphometric parameters of pancreas incretory apparatus in insulin resistant rats and after 2 weeks of treatment with dry bearberry leaves extracts

Experimental groups	Optical density (total Li number in microsample)	Li profile—the number of beta cells in the islet (distribution of pancreatic islets by beta cell content)									Functional index (β/α cells)
		Small			Medium			Large			
		Number	%	Perimeter, µm	Number	%	Perimeter, µm	Number	%	Perimeter, µm	
IC	19.83 ± 0.71	4.30 ± 0.81	21.77 ± 2.31	278.35 ± 30.23	13.33 ± 0.82	67.16 ± 2.85	861.31 ± 45.63	2.10 ± 0.75	10.83 ± 2.04	1935.13 ± 53.51	4.83 ± 0.41
Dex	16.50 ± 0.56*	5.83 ± 0.75*	35.35 ± 4.75*	139.55 ± 12.57*	8.33 ± 1.03*	50.61 ± 6.94*	718.11 ± 57.25*	2.33 ± 1.21	13.81 ± 4.91	2477.13 ± 385.37*	2.73 ± 0.44
Dex_PE50	17.72 ± 0.54^#^	5.79 ± 0.48	34.33 ± 1.54	262.80 ± 34.46#	8.83 ± 1.32	51.85 ± 6.17	809.75 ± 57.47#/&	2.16 ± 0.75	13.82 ± 3.14	1873.80 ± 85.55#	3.01 ± 0.12
Dex_PE50_cys	18.33 ± 0.51^#^	4.67 ± 0.51^#^	22.43 ± 2.62^#^	268.90 ± 37.08#	11.16 ± 0.98^#^	60.91 ± 5.19^#^	852.50 ± 55.87#	2.66 ± 0.51	14.52 ± 2.87	1767.85 ± 55.69*/#	3.26 ± 0.53
Dex_cys	16.95 ± 0.67*&	5.67 ± 0.54*	33.45 ± 0.46	157.48 ± 27.76*/&	9.74 ± 0.72*&	57.46 ± 4.67	735.51 ± 40.40*/&	2.03 ± 0.61	11.97 ± 3.21	1911.71 ± 62.45#	2.54 ± 0.23
Dex_arph	17.33 ± 1.03	5.83 ± 0.40	33.67 ± 1.21	232.20 ± 38.30*/#	9.33 ± 0.81	53.87 ± 3.86	802.66 ± 50.18&	2.16 ± 0.98	12.45 ± 5.24	1882.55 ± 71.19#	2.93 ± 0.39
Dex_met	18.66 ± 0.81^#^	4.66 ± 0.50^#^	24.97 ± 2.19^#^	293.63 ± 26.56#	11.50 ± 0.54^#^	61.65 ± 3.07^#^	880.50 ± 51.54#	2.50 ± 0.83	13.36 ± 4.50	1966.96 ± 82.12#	3.74 ± 0.38

Each value represents the mean ± standard error (n = 6)

^*^Indicates significant difference relative to IC group (p ≤ 0.05)

^#^Indicates significant difference relative to Dex group (p ≤ 0.05)

^&^Indicates significant difference relative to Dex_PE_cys group (p ≤ 0.05)

**Fig. 3:**
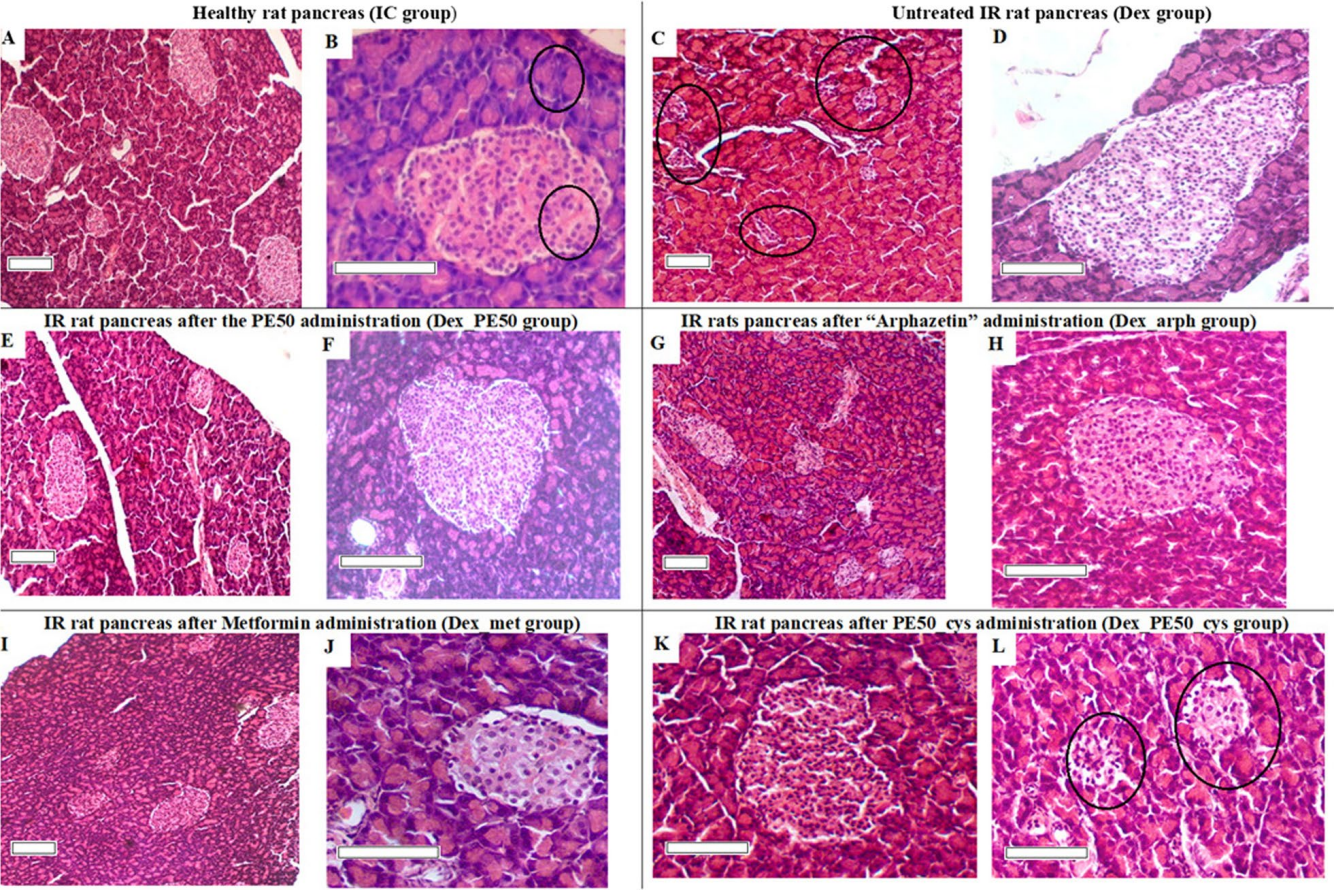
Photomicrograph of rat pancreas, H&E. **A** Normal size distribution of pancreatic islets (× 100). **B** Pancreatic acini and pancreatic beta cells are not changed (× 250). **C** Increased number of small pancreatic islets (× 100). **D** Very large pancreatic island (× 200). **E** Increase of the pancreatic islands average size (× 100). **F** Normalization of beta cells in the islet (× 200). **G** Increase in the pancreatic island size (× 100). **H** The pancreatic islands state reparation (× 200). **I** Visual restoration of the pancreatic islets number (× 100). **J** Some nuclear hypertrophy and beta cells cytoplasm vacuolation (× 250). **K** Unchanged pancreatic islet (× 200). **L** Vacuolation of beta cells and some devastation of the pancreatic island (× 200). Scale bar: 100 µm

After 7 weeks of dexamethasone intraperitoneal administration in Dex group, in contrast to IC group, there were fixed increased amount of small (by 1.6 times) and decreased medium in size (by 1.3 times) islets. In addition, the appearance of single extra-large Li was observed. Thus, the shape of the islands was mostly typical. The Li distribution by size was: small—35.37%, medium—50.61%, large 14.02%, respectively. The Li optical density was 16.50, because perimeter became significantly smaller, however, with a typical shape in small islands and in medium islands. In large Li, on the contrary, perimeter significantly increases, but the shape became «spread-eagled» (Table 4; Fig. 3).

The characteristic features of islet morphology in insulin resistance and overt diabetes are islet hypertrophy/hyperplasia and degeneration, respectively [30].

Based on the obtained histological data, the following summary can be made—IR modelling by administering daily dexamethasone low doses for 7 weeks leads to the morphological signs development of a certain insular apparatus depression.

Firstly, changes in endocrine part of pancreas after dexamethasone administration are manifested in an increase in the number of small pancreatic islets (5–20 beta-cells) and a decrease of medium (20–60 beta-cells) islets (Table 4). It can be assumed that the total number of beta cells in the islet apparatus is reduced, and the remaining ones are not able to meet the body's need for insulin, so some beta cells work in an enhanced mode. Morphologically, this is manifested in their hypertrophy and dystrophy. This, after a while, can lead to the depletion of such beta cells and, subsequently, to their death, which is expressed in varying degrees of devastation of insulin-producing cells in the pancreatic islet. The above morphological features inherent even to the diabetic “picture” [31]. The increase in the small islets proportion, from our point of view, reflects the body's response to islet atrophy, and, at the same time, show the certain adaptation processes including even acinar or duct epithelium cells transformation in the islet. Islet hypertrophy is also likely to reflect the compensatory-adaptive response of the insular apparatus to overexertion. Such islets contained mainly cells with small nuclei and a small volume of cytoplasm and may not have been functionally full-fledged.

After two weeks of PE50 and reference medication “Arphazetin” administration to IR rats were observed quite similar results. Thus, rather moderate increase in both the Li optical density and the renovation of islands distribution in size compared to control pathology—approximately 5–6% (Table 4). We fixed the positive effect of these combinations on the incretory apparatus in treating rats compared with the control pathology (Dex). Thus, in a notably larger number micropreparations Li were without dystrophic and destructive changes, although some islands still had these signs (Fig. 3). We did not observe actually large islets. No changes were detected in acinar tissue.

In rat’s pancreas under dexamethasone injections, PE50_cys and the reference medication Metformin administration also had a positive effect on the endocrine component of glandular tissue. The biggest part of Li had a typical shape, evenly filled with visually physiologically normal beta cells. No noticeable alpha cells proliferation was observed. Only in rare islands vacuolated beta cells and central zones devastation were found (Fig. 3). Focal perivascular and interacinar lymphocytic infiltration was observed in some rats in acinar tissue.

Treatment with PE50_cys from the 6th week of the experiment contributes to a certain inhibition of the dexamethasone effect on secretory apparatus. In insular tissue there are less indicative destructive-dystrophic manifestations, and morphologically healthier beta cells increase is visualized. To some extent, the pancreatic islets quantitative content and their percentage distribution by size (filled with beta cells) are restored. The effectiveness of PE50_cys administration in improving the endocrine tissue is slightly less than after Metformin administration, however, looks better in comparison with another reference medication—“Arphazetin”. The treatment with PE50 has a less indicative therapeutic effect than its combination with cysteine on the morphological state of the endocrine apparatus of the glandular tissue of rats in this model of pathology. We suppose that structural damage to pancreatic tissues as T2DM complications may be due to oxidative stress.

## Conclusions

The dry extracts were obtained from *Arctostaphylos uva-ursi* L. leaves with using cysteine. Their phytochemical profile, hypoglycemic and pancreatic protective effect were investigated.

Phenologlycoside (arbutin), phenolic carboxylic acid (gallic acid), 5 flavonoids and 4 hydroxycinnamic acids were identified and quantified in the bearberry extracts by HPLC. Hyperoside and catechin were dominant among flavonoids, and caffeic and chlorogenic acids were dominant among hydroxycinnamic acids.

Present data revealed that bearberry leaves alcoholic dry extract enriched with cysteine (PE50_cys) has a hypoglycaemic and pancreatic protective effect in treated animals under dexamethasone-induced IR model. Treatment by PE50_cys improved hyperglycaemia, insulin resistance and beta-cells reduction induced by dexamethasone. Further studies are required to determine the exact mechanism of PE50_cys on IR related changes in the pancreas.

## The experimental section

### Plant material

1 kg Leaves of *Arctostaphylos uva-ursi* L. (Spreng) were harvested in the Botanical Garden of Lviv Medical University named after Danylo Halytsky (49.83572429398166, 24.04946864960809). Voucher specimens no. 2017–2019 were deposited at the Department of Pharmacognosy (National University of Pharmacy, Kharkiv, Ukraine, No. 577–582). The identity of the plant was established with the consulting assistance of professor Tetiana Gontova, D.Sc. [32]. The raw material was dried at room temperature in a well-ventilated area for ten days and stored in paper bags.

### Preparation of extracts

The object of the study was a dry extract of bearberry leaves and its preparation with cysteine. 50 g of bearberry leaves was ground to a particle size of 2–3 mm and loss on drying was tested [33–35]. The raw material was placed in a flask, poured into 250 mL of a 50% solution of ethanol, and extracted overnight at room temperature. The extraction was repeated three times with new portions of the extractant (100 mL). The obtained extracts were combined, decanted during the day, filtered. The dry residue [33, 34] in the extract was 11.33%.

To 300 mL the combined extract was added cysteine (3.63 g), in three times the equimolar amount relative to the total amount of phenolic compounds in terms of gallic acid [36] and left for 24 h, after which the solutions were evaporated by rotary vacuum evaporator to dry extracts and ground (PE50_cys). The rest of the combined liquid extract was evaporated by rotary vacuum evaporator to dry extracts and ground (PE50). The temperature of the product in the final drying period was not higher than + 40 °C. The total duration of drying was 28–32 h.

### Analysis of the chemical composition of the extracts

*TLC analysis of the extracts.* Simple phenols were determined by ascending TLC in a system of solvents formic acid anhydrous–water–ethyl acetate (6:6:88). To do this, 0.05 g of extracts were dissolved in 5 mL of a mixture of equal volumes of methanol and water. The obtained extract solutions were used in subsequent TLC analyzes. For comparison, standard samples of gallic acid and arbutin dissolved in methanol at a concentration of 0.25% were used. Apply 10 μL of the test solution and 10 μL of the comparison solution. The chromatogram with the deposited substances was placed in a chamber with solvents. When the solvent front had passed about 15 cm, the plate was removed from the chamber and dried at 100–105 °C until traces of solvent were removed. Then the chromatogram was treated with a solution of 10 g/L of 4-aminopyrazolone P, then a solution of 20 g/L of potassium ferricyanide P and was shown in ammonia vapour [33, 37].

Hydroxycinnamic acids and flavonoids were detected by TLC in a system of organic solvents ethyl acetate–water–formic acid anhydrous–glacial acetic acid (72:14:7:7) in comparison with authentic samples of hydroxycinnamic acids (Sigma Chemical Company, USA). The presence of this group of compounds was detected by blue fluorescence in UV light after chromatogram treatment with reagents of 10 g/L of amyl ester of diphenylboronic acid in methanol and 50 g/L of macrogol 400 in methanol [33, 38].

Catechins in the extracts were determined by TLC, in accordance with the monograph of SPhU [33], in the solvent system, glacial acetic acid–ether–hexane–ethyl acetate (20:20:20:40). For comparison, standard samples of catechin and epigallocatechin dissolved in methanol at a concentration of 0.1% were used. Applied 20 μL of test solution and reference solution. The chromatogram with the applied substances was placed in a chamber with solvents. When the front of the solvents passed about 10 cm, the plate was removed from the chamber, dried in air to remove traces of solvents. Then the chromatogram was treated with a freshly prepared solution of 5 g/L of solid blue.

Taking into the account the toxicity of some substances, the presence of alkaloids was determined by reaction with Dragendorff’s reagent [39].

*Sample preparation, HPLC–DAD analysis and quantification*. 50.0 mg (exact weavage) of the bearberry extracts were weighed in a 5.0 mL measuring tube and brought to the mark with 90% aqueous methanol. After 30 min in an ultrasonic bath, the sample was insisted at room temperature for 3–4 h. Then the test tube was again placed on an ultrasound bath for 15 min, then the solution was filtered through a teflon filter with a pore size of 0.45 μm in vial for analysis [14, 40]. Standard substances manufactured by Sigma-Aldrich, USA were used for analysis.

Separation, identification and quantification of phenolic compounds were determined by HPLC using an Agilent Technologies chromatograph (model 1100) equipped with a vacuum degasser G1379A, four-channel low pressure gradient pump G13111A, automatic injector G1313A, G13116A column thermostat and G1316A diode-matrix detector. Columns 2.1 × 150 mm which was filled with octadecylsilyl sorbent grains of 3.5 μm "ZORBAX-SB C-18". The analysis of the bearberry extracts was carried out under the following conditions: thermostat temperature—35 °C; flow rate of the mobile phase—0.25 mL/min; as a mobile phase, solution A (0.1% H_3_PO_4_, 180 μL/L triethylamine, 3 mL/L tetrahydrofuran in water) and solution B (MeOH) in the ratio of 90:10 (first 8 min), 70:30 (from 8 for 24 min), and from 24 min only solution B was used; Working pressure of the eluent—240–300 kPa. In the analysis, the following detection parameters were set: scale of measurement—1.0; scan time—0.5 s; the parameters for removing the spectrum—each peak is 190–600 nm. Identification of phenolic compounds was performed by retention time of standards and UV spectral characteristics [16, 19].

The quantitative content of the basic groups of biologically active substances (BAS) in the bearberry extracts were determined by the method of absorption spectrophotometry on the spectrophotometer Evolution TM 60S UV–Visible (Thermo Fisher Scientific, USA) [16, 36]. In all the bearberry extracts the sum of the hydroxycinnamic acid derivates was determined by direct spectrophotometry (as chlorogenic acid, λ = 325 nm) (Koshovyi et al. [16]); flavonoids were quantified by the method of deferential spectrophotometry with aluminium chloride (as rutin, λ = 410 nm) [38]; polyphenols were quantified by direct spectrophotometry (as gallic acid, λ = 270 nm) [16, 36]. All assays were performed in triplicate.

### Study of the pharmacological activity of the extracts

#### Experimental groups

Male mature outbred albino rats were obtained from and housed in vivarium of Educational and Scientific Institute of Applied Pharmacy (National University of Pharmacy (NUPh), Kharkiv, Ukraine). Rats were fed standard diet and treated under conventional conditions with free access to water. In order to model experimental IR, we used low-dose dexamethasone (dex) injections (4 mg/L mL, KRKA, A 76532) [41]. Thus, according to the aim of experiment we randomized animals to the following groups (6 animals per group): 1—intact control animals (IC); 2—IR animals, which were injected intraperitoneally daily by dex (KRKA, Slovenia) 15 mkg/kg bw for 7 weeks (Dex); 3—IR animals (see group Dex), which beginning from 5th week of experiment were administered per os PE50 in dose 100 mg/kg bw for 2 weeks (Dex_PE); 4—IR animals (see group Dex), which beginning from 5th week of experiment were administered per os PE50_cys in dose 100 mg/kg bw for 2 weeks (Dex_PE_cys); 5 – IR animals (see group Dex), which beginning from 5th week of experiment were administered per os cysteine (l-Cysteine, Sigma-Aldrich) in dose 100 mg/kg bw for 2 weeks (Dex_cys); 6 – IR animals (see group Dex), which beginning from 5th week of experiment were administered per os Metformin in dose 100 mg/kg bw for 2 weeks (Dex_met); 7 – IR animals (see group Dex), which beginning from 5th week of experiment were administered *per os* “Arphazetin” tea infusion in dose 18 mL/kg bw for 2 weeks (Dex_arph).

#### Pancreatic oxidative stress markers

On the last day of the experiment the fasting for 12 h rats were sacrificed by decapitation and blood samples were collected to obtain the blood serum. Pancreas were excised, perfused, waited and rinsed in ice-cold physiological saline. 10% (w/v) homogenates (10 mM Tris–HCl-buffer pH 7.4) were prepared with the help of plastic-coated Potter Elvehjem homogenizer. To evaluate oxidative stress in pancreatic tissue homogenates were determined thiobarbituric acid reactive substances (TBARS) [42], diene conjugates (DC) [43] and reduced glutathione (GSH) [44] levels as well as tissue activity of superoxide dismutase (SOD) [45], glutathione peroxidase (GPx) [46] and catalase (CAT) [47] (Tenovuo, 1986) activity according to the unified methods pointed in square brackets. Protein concentration in homogenates was determined by the method of Lowry J.Y Lowry in G.L. Miller modification [48], using bovine serum albumin as the standard. In pancreas homogenate total JNK was determined with the help of commercial Human/Mouse/Rat Total JNK Pan Specific DuoSet IC ELISA (R&D Systems, Inc., USA). Phospho-JNK1/2 (p-JNK) in pancreas was determined with the help of commercial [pThr183/Tyr185] JNK1/2 EIA kit (Enzo Life Sciences). Fasting blood glucose (FBG) and immunoreactive insulin (IRI) concentration were determined using commercially available kits (“Felicit Diagnostics”, Ukraine and “DRG”, Germany, respectively). And then calculated homeostasis model assessment (HOMA) index using calculator Oxford website (https://www.dtu.ox.ac.uk/homacalculator/: HOMA-IR = (10 × G0)/22.5, where G—FBG in mmol/L).

#### Histopathological changes in the pancreas

For histopathology examination representative fragments of the pancreas were fixed in a 10% solution of formalin, rehydrated by different graded ethanol dilution and enclosed in paraffin. The obtained tissue sections were then mounted on glass slides, coloured with Haematoxylin–Eosin (H&E). All slides were examined using light microscopy Granum equipped with a digital camera Granum DCM 310. On sections of the pancreas determined the Li optical density—the total number of pancreatic islets in microsample, Li profile—the number of β-cells in the islet, and functional index—ratio of beta cells to alpha cells. The perimeter of the pancreatic islets (µm) was measured using the Toupcam Granum software. According to the indicator of the Li profile, the islands were divided on the basis of islet size into small (≤ 20 beta cells), medium (21–60 beta cells) and large (> 61 beta cells), determined the percentage of each category of pancreatic islets.

### Statistical analysis

Statistical properties of random variables with n-d dimensional normal distribution are given by their correlation matrices, which can be calculated from the original matrices. Statistical assessment all pharmacological data are reported as mean ± SEM and were analysed using STATISTICA 6 software with one-way ANOVA. P values less than 0.05 was assumed statistically significant [33, 49, 50].

All procedures and euthanasia were carried out in compliance with the principles of the “European Convention for the Protection of Vertebrate Animals Used for Experimental and Scientific Purposes” (Strasbourg, 1986) and were approved by the Ethics Committee for Animal Experiments of the NUPh (Protocol #3 from 10.09.2020; Approval #3/10092020). The study was conducted according to the guidelines of the Declaration of Helsinki, and approved by the Ethics Committee of the National University of Pharmacy (approval #3/10092020). The experiment was carried out in accordance with the International Principles of the European Convention for the Protection of Vertebrate Animals Used for Experimental and Other Scientific Purposes.
